# Gene Expression Profile of Increased Heart Rate in Shensongyangxin-Treated Bradycardia Rabbits

**DOI:** 10.1155/2014/715937

**Published:** 2014-11-27

**Authors:** Zhouying Liu, Jian Huang, Roumu Hu, Youping Huo, Jing Gong, Yinhui Zhang, Cong Wei, Jielin Pu

**Affiliations:** ^1^State Key Laboratory of Translational Cardiovascular Medicine, Fuwai Hospital & Cardiovascular Institute, Chinese Academy of Medical Sciences and Peking Union Medical College, 167 Bei-Li-Shi Road, Xi-Cheng District, Beijing 100037, China; ^2^Integration of Traditional and Western Medical Research Academy of Hebei Province, Shijiazhuang 050035, China

## Abstract

*Aims*. The present study tries to investigate the gene expression profile of bradycardia rabbits' hearts after SSYX (SSYX, a traditional Chinese medicine) treatment. *Methods*. Eighteen adult rabbits were randomly assigned in three groups: sham, model, and SSYX treatment groups. Heart rate was recorded in rabbits and total RNA was isolated from hearts. Gene expression profiling was conducted and quantitative real-time reverse transcription-polymerase chain reaction (RT-PCR) was performed to confirm the gene expression results. Patch clamp using human induced pluripotent stem cell-derived cardiomyocytes was applied to record the calcium current in the presence of SSYX. *Results*. The mean RR interval reduced after six weeks due to the injury of the sinoatrial node in the model group. This effect was partially reversed by 4-week SSYX treatment. cDNA microarray demonstrated that genes related with pacemaker current, calcium ion homeostasis, and signaling were altered by SSYX treatment. Results from patch clamp demonstrated that SSYX reduced the calcium current which is consistent with gene expression results. *Conclusion*. The present study shows mRNA remodeling of bradycardia and demonstrates that SSYX is effective in treating bradycardia by reversing altered gene expression in bradycardia models. Reduced calcium current by SSYX also confirmed the gene expression results.

## 1. Introduction

Bradycardia is a condition in which pulse rate is below 60 beats per minute (bpm). Coronary artery disease patients and elderly people are at a great risk of developing the abnormally slow heart rate [1]. Currently available drugs (e.g., atropine, dopamine, isoproterenol, and epinephrine) treating bradycardia are temporizing measures only in emergency settings. If the patient does not respond to drugs, temporary or permanent cardiac pacemaker is probably indicated [2]. However, the cost of pacing put a huge financial burden on the family. Consequently, an effective drug aiming at increasing heart rate for a long term is in urgent demand.

Traditional Chinese medicines have been used to treat arrhythmia for hundreds of years and Shensongyangxin (SSYX) is one of such medicines. It is a product consisting of 12 ingredients including* Panax ginseng*, dwarf lilyturf tuber, and* Nardostachys* root. Whole-cell patch clamping experiments revealed that SSYX could block multiple ion channels [3]. Preliminary studies also suggested that SSYX was effective in reducing ventricular premature beat and treating bradycardia [4, 5]. In addition, the latest results from a randomized, double-blind, placebo-controlled multicenter trial demonstrated that SSYX is effective for patients with bradycardia [6]. However, it has been a mystery how SSYX plays the positive role in treatment. Therefore, we established an animal model of bradycardia and explored the gene expression profiling of heart after SSYX treatment. Our work will provide new insights into the molecular mechanisms of SSYX.

## 2. Materials and Methods

### 2.1. Bradycardia Model

Eighteen adult rabbits (*Oryctolagus cuniculus) *with mean body weight of 2.5 ± 0.5 kg were used for the study. The experimental protocol was performed in accordance with the Guide for the Care and Use of Laboratory Animals (NIH Publication number 85-23, revised 1985) and The ARRIVE [7]. The Care of Experimental Animals Committee of the Chinese Academy of Medical Sciences and Peking Union Medical College approved the procedures for the care and treatment of animals. The animals were randomly divided into three groups (*n* = 6 in each group): sham, model, and model plus SSYX (SSYX) groups. Sterilized cotton bud with formaldehyde (37%, SCRC) was fixed on the wall of the right atrium, near the entrance of the superior vena cava until heart beat decreased 25–35% [8]. The time span of the process was ranged from 20 seconds to 3 minutes. Continuous monitoring by electrocardiogram for two weeks confirmed the slow heart rate. Then, purified water was administered orally to model group while dry powder of SSYX (220 mg/kg/d) dissolved in purified water was administered to SSYX group. Lead II was used to monitor the electrocardiogram, once every week for 4 weeks. At last, animals were sacrificed 6 weeks later. The procedures in sham group were similar to model and SSYX group except that formaldehyde was replaced by purified water. RR, P, PR, QRS, QT, and QTc were calculated before operation (baseline) and 6 weeks later, respectively.

### 2.2. RNA Preparation

Hearts of the animals were isolated and perfused with purified water. Atria and ventricle of the heart were immediately frozen in liquid nitrogen and then stored at −80°C until use for RNA extraction. MirVana mRNA isolation kit (Ambion-1561) was used in accordance with the manufacturer's instructions to isolate total RNA. Then, NanoDrop ND-2000 (Thermo Scientific) and Agilent Bioanalyzer 2100 (Agilent Technologies) were used to quantify RNA and assess the RNA integrity, respectively. To minimize variations attributable to individual rabbit and maximize differences attributable to their genotype, each experiment was performed with RNA pooled from 3 atria.

### 2.3. Gene Expression Profiling

Total RNA was transcribed to double strand cDNA, then synthesized into cRNAs, and labeled with Cy3. Labeled cRNAs were hybridized onto Agilent Rabbit Gene Expression Chip (4∗44K, Design ID: 020908, containing 43,803 probes) according to manufacturer's instructions and scanned by Agilent Scanner G2505C (Agilent Technologies). Feature Extraction software (version 10.7.1.1, Agilent Technologies) was used to analyze array images to get raw data. Data normalization was performed using Genespring. Differentially expressed genes were selected based on fold change greater than 2.0 and removed using differentially expressed genes filtering. Gene ontology database and KEGG were applied to determine functions of these differentially expressed mRNAs [9]. Then, differentially expressed genes were grouped by Funnet according to biological process classification. The statistical significance of the gene enrichment was assessed by Fisher's exact test. 5% false discovery rate (FDR) was employed for controlling statistical errors. The most increased and decreased gene groups were selected on the basis of significance analysis of *P* < 10^−4^. To obtain the gene expression matrix consisting of differentially expressed genes, a two-way hierarchical agglomerative clustering was applied using cluster, average linkage clustering was applied with uncentered correlation, and clusters were visualized using TreeView.

### 2.4. Quantitative PCR

SYBR Green quantitative real-time reverse transcription-PCR (RT-PCR) was performed on the genes BDNF, FN1, TBX20, KCNJ11, ERBB2, GUCY1B3, PRKG1, and 18S rRNA (as an internal control) to confirm the results of gene expression chip. Genes were selected from interesting functional groups revealed by gene ontology analysis. Primers were designed with LightCycler Probe Design software 2.0 (Roche Applied Bioscience) (Table 2). The cDNA was synthesized at 37°C for 15 min in a 10 *μ*L reaction containing 0.5 *μ*g total RNA, 2 *μ*L PrimeScript Buffer, 0.5 *μ*L oligo dT, 0.5 *μ*L random 6 mers, and 0.5 *μ*L PrimeScript RT Enzyme Mix I (TaKaRa). Real-time RT-PCR reactions included 1 *μ*L of cDNA, 5 *μ*L 2× LightCycler 480 SYBR Green I master mix (Roche), 0.2 *μ*L forward primer, 0.2 *μ*L reverse primer, and 3.6 *μ*L nuclease-free water. All PCR reactions were carried out in triplicate with the following conditions: 95°C for 10 min, followed by 40 cycles of 10 s at 95°C, 30 s at 60°C in the LightCycler 480 II real-time PCR Instrument (Roche). For each selected gene, melting curve analysis was performed to validate the specific generation of the expected PCR product. The expression of each gene was normalized as ΔC_t_ (C_t_ of target gene—C_t_ of internal control gene) using 18S rRNA as the control. Relative quantification using the ΔΔC_t_ method was applied to compare the amounts of mRNA in sham versus model groups and model versus SSYX groups [10].

### 2.5. Electrophysiological Recording

Human induced pluripotent stem cell-derived cardiomyocytes (Cellapy, China) were used to confirm result of microarray. The whole-cell patch clamp technique was used to record *I*_Ca-L_ using an Axopatch 700B amplifier (Axon Instruments, USA) with the pCLAMP 10.0 software (Axon Instruments, USA). Borosilicate glass patch pipettes (tip resistance of 2-3 MΩ) were pulled using a horizontal pipette puller (P-97, Sutter Instrument, USA). Cells were superfused with Tyrode's solution supplemented with 1.8 mmol/L CaCl_2_ including 0.5% SSYX for 3 min. The internal pipette solution contained the following (in mmol/L): 120 CsCl, 1.0 CaCl_2_, 5.0 MgCl_2_, 5.0 Na_2_ATP, 11 EGTA, 10 HEPES, and 11 glucose (pH 7.3 with CsOH). All of the recordings were performed at room temperature (22°C). The *I*_Ca-L_ was recorded in the voltage-clamp mode and elicited by step depolarization from −40 to +60 mV in 10 mV increments for 250 ms followed by a repolarization from +40 to −60 mV. A paired-pulse protocol was used to evaluate the recovery from inactivation of *I*_Ca-L_: a conditioning pulse was first applied from a holding potential of −70 mV to +10 mV, and a test potential of 10 mV for 250 ms was then applied after various interval durations of 1, 3, 5, 8, 12, 20, 40, 60, 100, 160, 300, 500, and 1000 ms. Boltzmann function was used to obtain voltage dependence of channel activation. Origin 8.0 software (Microcal Software, USA) was used for the data analysis. Cells in the absence and presence of 0.5% SSYX are the same cells. The data are presented as the mean ± SE; 5 cells were analyzed.

### 2.6. Statistical Analysis

Electrocardiogram data were expressed as mean ± SEM. Two-way ANOVA was used to test difference of basic parameters between groups. Independent sample *t*-test was used to estimate difference between groups, with *P* < 0.05 considered as significant. Analyses were performed with SPSS 17.0 (SPSS lnc., Chicago, IL).

## 3. Results

### 3.1. Effect of Long-Term SSYX Treatment on Slow Heart Rate

**Figure 1:**
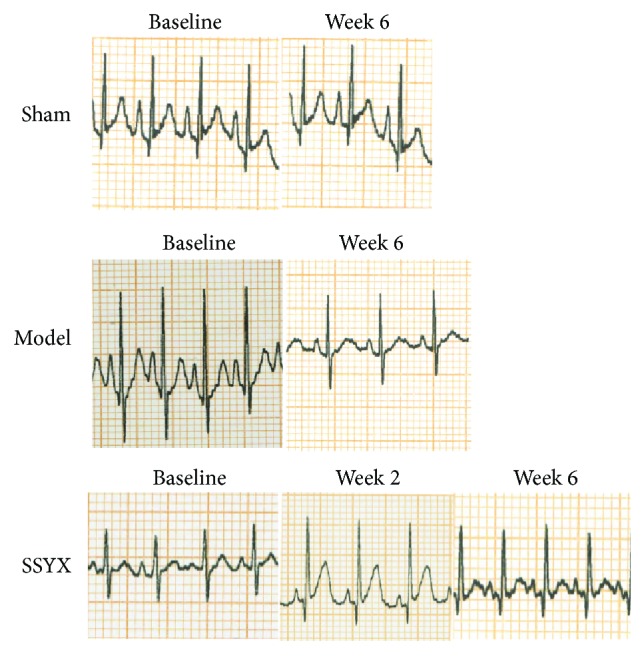
SSYX effects on cardiac electrical activity in anesthetized rabbits. Representative ECG recording (lead II) obtained in one rabbit from sham (top), model (middle), and SSYX group (bottom) under baseline conditions (previous to operation) and after 2 weeks and 6 weeks of treatment.

Representative ECG recordings of sham, model, and SSYX-treated rabbits are illustrated and analyzed in Figure 1 and Table 1, respectively. No difference was observed among baselines of the three groups (*P* > 0.05). As is evident, chemical injury of sinoatrial node increased constantly the mean RR interval by 32% (from 275 ± 17 in sham group to 406 ± 35 in model group, *P* < 0.05, *n* = 6, resp.) after six weeks. This effect was partially reversed by 4-week SSYX treatment (220 mg/kg*·*d, from 406 ± 35 in model group to 268 ± 20 in SSYX group, *P* < 0.05). SSYX had no significant effect on atrial, atrioventricular, and ventricular conduction parameters, since the P, PR, and QRS were not modified. In addition, ventricular repolarization was also not affected because of unmodified corrected QT interval (Table 1).

### 3.2. Effects of Long-Term SSYX Treatment on Cardiac Transcripts

**Figure 2:**
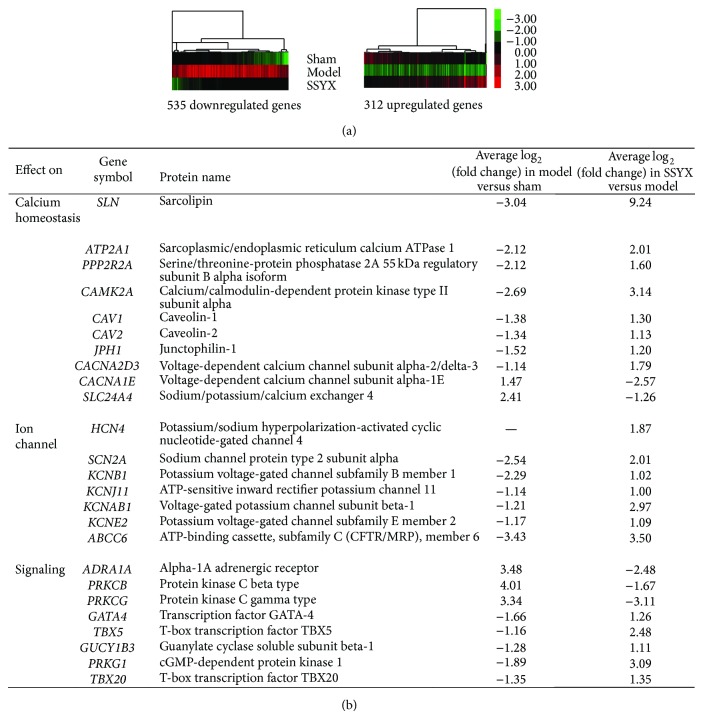
Altered expressed genes in model versus SSYX treatment group and SSYX treatment versus model group. (a) Hierarchical clustering of differentially expressed genes by cluster. Gene expression profiles of SSYX effect on pathological rabbits were executed according to a 2-fold change cutoff. A total of 846 genes deregulated, among which 535 were downregulated and 311 were upregulated. (b) A list of altered genes of interest involved in calcium homeostasis, ion channel, and signaling.

To verify the gene expression changes induced by chemical lesions of SA node, we compared sham and model group and identified 1940 altered genes, among which 1049 genes were downregulated (named as lesion-Down) and 891 were upregulated (named as lesion-Up). In order to follow the changes induced by SSYX treatment, we compared model and SSYX-treated rabbits and found 1689 differentially expressed genes (799 downregulated (SSYX-Down) and 890 upregulated (SSYX-Up)). All altered genes were displayed in Supplement Table 1 (see Supplementary Material available online at http://dx.doi.org/10.1155/2014/715937). Supplement Table 2 showed 846 restored genes between SSYX-Down versus lesion-Up and SSYX-Up versus lesion-Down.  Figure 2 displayed an overview of restored expressed genes through hierarchical clustering analysis (Figure 2(a)).

Finally, as shown in Supplement Figure 1, restored genes were involved in muscle contraction, cellular calcium ion homeostasis, cardiac muscle fiber development, mitotic cell cycle, cell division, and DNA replication, mitosis, and so forth.

According to our results, long-term SSYX treatment restored the normal heart rate. In this way, differentially expressed genes induced by SSYX treatment tried to restore the altered gene expression induced by lesion on SA node. Among these restored genes, three groups of genes related to calcium homeostasis, ion channel, and signaling were the most of interest (Figure 2(b)). Calcium current is important in the phase 4 of action potential in SA node. Therefore, eight restored genes related to calcium homeostasis played an important role in the increased heart rate. Transporters such as ATP2A1, CACNA2D3, and SLC24A4 and regulators PPP2R2A, CAMK2A, JPH1, and CAV1/2 were included in this group. Increased S100B after SSYX treatment might also contribute to the restoration of calcium homeostasis. Multiple signal pathways might also account for the restored heart rate. For example, restored adrenergic receptor (ADRA1A) and PKC (PRKCB, PRKCG) indicated the restored signaling mediated by adrenergic receptor while GATA4-TBX5 signaling plays an important role in cardiomyocytes survival [11]. Previous studies showed that TBX20 is also associated with heart rate [12, 13]. Most importantly, the expression of HCN4 is increased. HCN4 encodes the potassium/sodium hyperpolarization-activated cyclic nucleotide-gated channel 4 and is responsible for the phase 4 depolarization of the action potential in cardiomyocytes. It contributes to the native pacemaker currents in heart (*I*_*f*_) that determine the heartbeat [14].

### 3.3. Verification of Altered Gene Expression by Quantitative Real-Time RT-PCR

Quantitative real-time RT-PCR was performed to confirm the results from gene expression chip. Seven altered genes, BDNF, FN1, TBX20, KCNJ11, ERBB2, GUCY1B3, and PRKG1, were selected. For example, TBX20 was chosen for further study because knockdown of this gene could lead to slower heart rate [12, 13]. The relative mRNA expression level of each selected gene was normalized to that of 18S rRNA. As demonstrated in Supplement Figure 2, the relative expression of these genes was decreased in model versus sham. Furthermore, the relative expression level of them increased after four weeks treatment with SSYX. Therefore, the trend of mRNA expression changes as verified by real-time RT-PCR was in agreement with that detected by gene expression chip (Supplement Figure 2). 

### 3.4. Effects of SSYX on the *I*_Ca-L_

**Figure 3:**
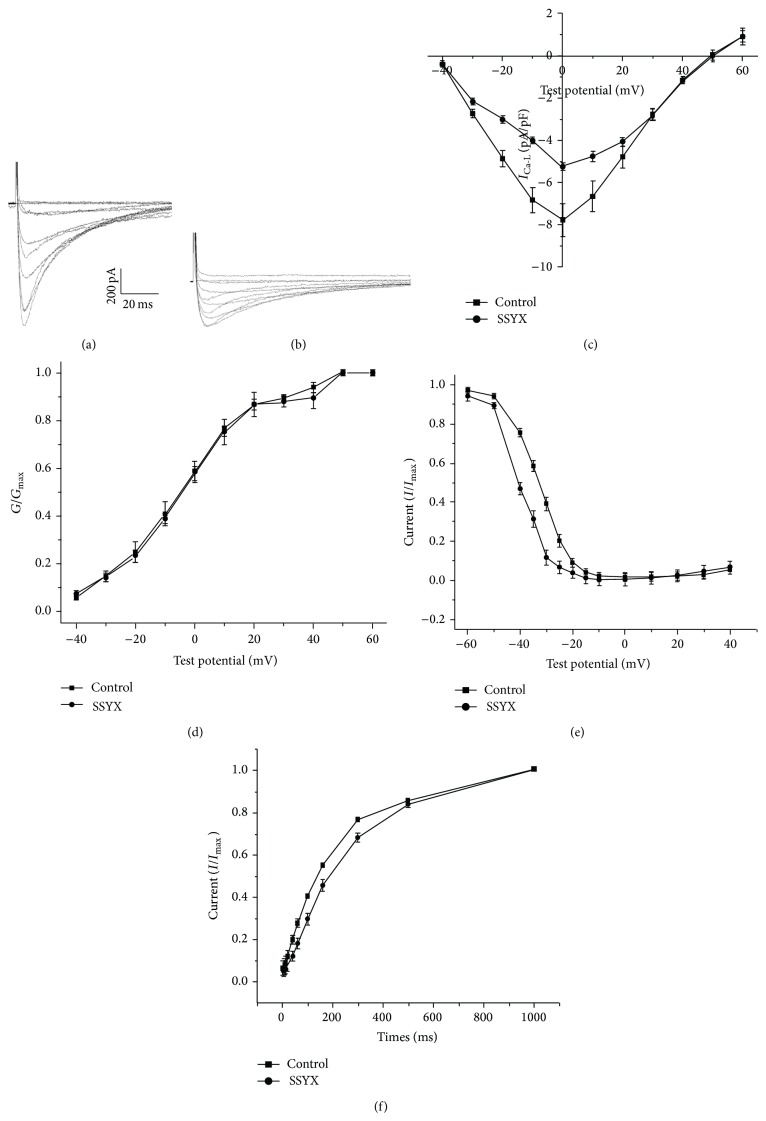
Effect of SSYX on the *I*_Ca-L_ in human induced pluripotent stem cell-derived cardiomyocytes. Representative *I*_Ca-L_ traces recorded from the absence (a) and presence of 0.5% SSYX (b), which showed the reduced current. (c) The peak current density-voltage relationship from two groups. The current density at 0 mV was decreased from −7.77 ± 0.76 pA/pF in the model group to −5.25 ± 0.11 pA/pF after SSYX administration (*n* = 5, *P* < 0.05). The (d) steady-state activation curve, (e) inactivation curve, and (f) recovery curve in model and SSYX groups. The left shifted steady-state inactivation curve (*n* = 5, *P* < 0.05) demonstrated that SSYX delayed the recovery time from inactivation. The data are presented as the mean ± SE; five cells were analyzed. Cells in the absence and presence of 0.5% SSYX are the same cells.

The voltage-dependent *I*_Ca-L_ traces as shown in Figure 3 recorded the absence (Figure 3(a)) and presence (Figure 3(b)) of 0.5% SSYX in human induced pluripotent stem cell-derived cardiomyocytes. The reduced peak current was clearly displayed. To avoid the effect of current rundown, all the recordings were obtained within 15 min.  Figure 3(c) shows the current-voltage (*I*-*V*) correlation for *I*_Ca-L_; it suggests that SSYX markedly reduced the amplitude of *I*_Ca-L_. The current density at 0 mV was decreased from −7.77 ± 0.76 pA/pF in the model group to −5.25 ± 0.11 pA/pF after SSYX administration (*n* = 5, *P* < 0.05).

The steady-state activation curve exhibited no significant difference between groups (Figure 3(d)). SSYX treatment moderately shifted the half-inactivation potential (*V*_1/2,act_) from 5.3 ± 1.38 mV in model group to 4.45 ± 1.97 mV (*n* = 5, *P* > 0.05). The slope factor (*k*_act_) activation values in model group and SSYX group were 14.15 ± 1.22 mV and 13.34 ± 1.81 mV, respectively (*n* = 5, *P* > 0.05). In the presence of SSYX, the steady-state inactivation curve was shifted to the left (Figure 3(e)). The half-inactivation potentials in model and SSYX groups were 32.97 ± 0.30 mV and −39.92 ± 0.62 mV (*n* = 5, *P* < 0.05). The slope factor (*k*_inact_) inactivation values in model and SSYX groups were 5.47 ± 0.26 mV and 4.83 ± 0.52 mV (*n* = 5, *P* < 0.05). These results revealed that SSYX reduced the current by accelerating inactivation of the channels. The treatment with SSYX shifted the recovery curve from the inactivation of *I*_Ca-L_ in model group to the right (Figure 3(f)). The results demonstrated that SSYX delayed the recovery time from inactivation.

## 4. Discussion

Microarray technology has been applied to explore transcriptome alterations in cardiovascular diseases such as atrial fibrillation and heart failure [15, 16]. Two studies which investigated reduced heart rate effect of Amiodarone and Ivabradine also obtained gene expression profiling in heart rate disorders [17, 18]. Both studies focused on ion channel remodeling between model and treatment group. However, mRNA expression profiling of bradycardia has not been obtained. The present study shows directly or indirectly mRNA remodeling of bradycardia for the first time and demonstrates that SSYX is effective in treating bradycardia. However, it is not possible to assume that all changes in gene expression are coupled to the development of bradycardia. In fact, the present data did not exclude the possibility that the part of the gene expression modifications was associated with the SA lesion or secondary to the development of bradycardia.

### 4.1. Comparison with Previous Study

In contrast to Amiodarone and Ivabradine, SCN2A and KCNB1 were upregulated in SSYX-treated rabbits, which indicate accelerated conduction and shortened repolarization. In another word, the expression level of SCN2A and KCNB1 was altered in both normal and bradycardia animals; their expression level increased in accelerated heart rate whereas it decreased in case of slow heartbeat. Kir6.2 (KCNJ11) forming ATP-sensitive inward rectifier potassium channel and decreasing in Amiodarone-treated animals was overexpressed in SSYX-treated rabbits. In contrast, Kv*β*1 (KCNAB1) and MiRP1 (KCNE2), encoding K^+^ channel regulatory subunits and increased in Amiodarone-treated mice, were also overexpressed in SSYX-treated rabbits [17]. Overexpression of voltage-gated Ca^2+^ channel subunit alpha-2/delta-3 (CACNA2D3) was obtained in both Ivabradine- and SSYX-treated animals [18]. Different animal models may account for the upregulated expression level of KCNAB1, KCNE2, and CACNA2D3. In both previous studies, instead of setting up a pathological animal model, normal C57BL/6 mice were supplied with either Amiodarone or Ivabradine. However, the present study explored the mechanisms of SSYX on bradycardia rabbit.

### 4.2. Potential Mechanisms for SSYX-Induced mRNA Remodeling

The molecular mechanisms of SSYX administration have principally been unknown. Prior to the study, we hypothesized that SSYX might affect the potassium/sodium hyperpolarization-activated cyclic nucleotide-gated channel 4 (HCN4) or activate the adrenoceptor mediated signaling. In fact, the overexpression of HCN4 and decreased expression of AC (ADCY2)-PKA (PRKACA, PRKACB), *α*-AR (ADRA1A), and PKC (PRKCB, PRKCG) were detected after SSYX treatment. These results suggested that SSYX increased pacemaker currents (*I*_*f*_) without activating adrenoceptor mediated signaling. Previous acute study has shown that SSYX block pacemaker currents shortly after adding SSYX [19]. This suggests that SSYX may act differentially according to different time span.

Furthermore, altered genes such as ATP2A1, SLC, PPP2R2A, CAMK2A, CAV1/2, JPH1, SLN, and S100B in SSYX-treated animals suggest that SSYX also make efforts to restore calcium homeostasis. It is worth noting that the pore-forming and voltage-sensitive alpha-1 subunit of L-type calcium channel (CACNA1E), which determines the channel activity, was increased after the lesion on SA node. It indicates that the lesion probably leads to the enhanced calcium current. Moreover, its expression decreased after SSYX treatment, which means the reduced calcium current. Therefore, we studied the calcium current in the absence and presence of SSYX. According to the results of patch clamping experiments, SSYX acutely reduced the calcium current by accelerating inactivation of the channels and delayed the recovery time from inactivation. Although it was an acute experiment, the result is in consistent with the chronic result.

Transcription factor TBX20 is an interesting candidate gene that probably contributed to the increased heart rate, because certain previous research has demonstrated that* Drosophila* and adult mice with TBX20 knockout showed slow heart rate [12, 13]. Overexpressed GATA4 (a zinc finger-containing transcription factor) and its cooperator-TBX5 are also worth noting. GATA4 plays an essential role in promoting cardiomyocytes survival in the adult heart [11]. On the other hand, TBX5-encoded protein plays a role in heart development and affects heart conduction system [20]. Hence, their overexpression can probably lead to cardiomyocytes survival and a better differentiation and also can give rise to a better conduction system. It has also been found that cell proliferation is promoted through overexpressed ERBB2/4 (member of the epidermal growth factor receptor subfamily) and HBEGF which is located in the upstream of ERBB2/4. Those overexpressed genes may activate downstream MAPK cascade to regulate cell growth.

Finally, SSYX probably affect the relaxation of vascular smooth muscle, which is regulated by NO/cGMP/PKG pathway. Overexpression of cGMP (GUCY1B3) and PKG (PRKG1) probably leads to the relaxation of vascular smooth muscle, which improves blood supply of cardiomyocytes.

Our bradycardia model showed that long-term SSYX treatment increased heart rate by reversing a wide range of genes. It restored the expression of genes related to muscle contraction, calcium ion homeostasis, cell proliferation, and growth factor activity. HCN4, TBX20, and so forth were the most potential candidate genes contributing to the effect of SSYX treatment. In addition, path clamp results also confirmed the reduced calcium current which is in consistent with results from gene expression. These data provide insights for the future study of SSYX.

## Supplementary Material

Supplement Tables showed all altered genes after SSYX treatment (Table 1) and showed 846 restored genes between SSYX-Down versus lesion-Up and SSYX-Up versus lesion-Down (Table 2). Supplement Figure 1 showed Gene ontology classification of differentially expressed genes after SSYX treatment. Representation of biological process items obtained from the deregulated genes after SSYX treatment, (A) downregulated and (B) upregulated (according to a unilateral Fisher exact test and P < 10-4). Supplement Figure 2 displayed the results from quantitative real-time RT-PCR. (A) Comparison of results obtained with the expression chip and quantitative real-time RT-PCR for mRNA levels of selected genes in sham vs. model group and (B) in SSYX vs. model group.

## Figures and Tables

**Table 1:** ECG parameters in anesthetized rabbit from sham, model, and SSYX groups.

	RR, ms		P, ms		PR, ms		QRS, ms		QT, ms		QTc, ms	
	Baseline	Week 6	Baseline	Week 6	Baseline	Week 6	Baseline	Week 6	Baseline	Week 6	Baseline	Week 6
Sham	280 ± 17	275 ± 17	40 ± 0	40 ± 0	63 ± 3	63 ± 3	23 ± 3	23 ± 3	170 ± 4	170 ± 4	102 ± 3	103 ± 2
Model	241 ± 10	406 ± 35^*※*^	40 ± 0	40 ± 0	62 ± 1	60 ± 0	22 ± 2	25 ± 3	163 ± 3	185 ± 11	105 ± 2	93 ± 6
SSYX	233 ± 8	268 ± 20^*^	40 ± 0	37 ± 3	63 ± 3	56 ± 3	30 ± 4	27 ± 4	148 ± 4	160 ± 12	97 ± 2	92 ± 4

Data expressed as mean ± SEM; *n* = 6 in each group. Electrocardiogram parameters obtained in anesthetized rabbit from sham, model, and SSYX groups before treatment (baseline) and after 6 weeks of treatment. RR: RR interval; P: P wave duration; PQ: PQ interval; QRS: QRS complex duration; QT: QT interval; QTc: corrected QT interval; QTc = QT/2√(RR/100).

^*※*^ *P* < 0.05 versus sham group.

^*^ *P* < 0.05 versus model group.

**Table 2:** Primers for quantitative real-time RT-PCR.

Gene symbol/GeneBank	Primer	Sequence (5^'^→3′)	Amplified length (bp)
BDNF (XM_002709025)	Forward Reverse	CTGTTGGATGAGGACCAGA GGCTCCAAAGGCACTTGA	104

FN1 (XM_002712573)	Forward Reverse	TCTGGCTTTAAGCTCTCGT ATCTTGTAGTTCACACCGT	101

TBX20 (XM_002713752)	Forward Reverse	AGAGCCTGATTCAGAAGC CCAGGAACTGAGAGACAAATTA	146

KCNJ11 (NM_001082017)	Forward Reverse	CACCTCCTACCTGGCAGA TTGCCAAACTTGGAGTAGTC	102

ERBB2 (XM_002719343)	Forward Reverse	CGTCAAGATCACAGACTTCG GGCGGAGAATGGACTCCAA	116

GUCY1B3 (XM_002716886)	Forward Reverse	AAGACAGACACATTGCTGTA GAAGAGGATGGTCACGTT	108

PRKG1 (NM_001082042)	Forward Reverse	TTACACAGCATGTGTGGTAG AATTTAGCATAACCTCGGTGA	111

18S rRNA	Forward Reverse	CGGCTACCACATCCAAGGAA GCTGGAATTACCGCGGCT	187
